# Diverging functional connectivity timescales: Capturing distinct aspects of cognitive performance in early psychosis

**DOI:** 10.1016/j.nicl.2024.103657

**Published:** 2024-08-23

**Authors:** Fabian Hirsch, Ângelo Bumanglag, Yifei Zhang, Afra Wohlschlaeger

**Affiliations:** Department of Diagnostic and Interventional Neuroradiology, Klinikum R.d.Isar, Technical University Munich, Ismaninger Str. 22, Munich 81675, Germany

**Keywords:** fMRI, Resting-state, Time-varying connectivity, Psychosis, Cognition, Neurotransmitters

## Abstract

**Background:**

Psychosis spectrum disorders (PSDs) are marked by cognitive impairments, the neurobiological correlates of which remain poorly understood. Here, we investigate the entropy of time-varying functional connectivity (TVFC) patterns from resting-state functional magnetic resonance imaging (rs-fMRI) as potential biomarker for cognitive performance in PSDs. By combining our results with multimodal reference data, we hope to generate new insights into the mechanisms underlying cognitive dysfunction in PSDs. We hypothesized that low-entropy TVFC patterns (LEN) would be more behaviorally informative than high-entropy TVFC patterns (HEN), especially for tasks that require extensive integration across diverse cognitive subdomains.

**Methods:**

rs-fMRI and behavioral data from 97 patients in the early phases of psychosis and 53 controls were analyzed. Positron emission tomography (PET) and magnetoencephalography (MEG) data were taken from a public repository (Hansen et al., 2022). Multivariate analyses were conducted to examine relationships between TVFC patterns at multiple spatial scales and cognitive performance in patients.

**Results:**

Compared to HEN, LEN explained significantly more cognitive variance on average in PSD patients, driven by superior encoding of information on psychometrically more integrated tasks. HEN better captured information in specific subdomains of executive functioning. Nodal HEN-LEN transitions were spatially aligned with neurobiological gradients reflecting monoaminergic transporter densities and MEG beta-power. Exploratory analyses revealed a close statistical relationship between LEN and positive symptom severity in patients.

**Conclusion:**

Our entropy-based analysis of TVFC patterns dissociates distinct aspects of cognition in PSDs. By linking topographies of neurotransmission and oscillatory dynamics with cognitive performance, it enhances our understanding of the mechanisms underlying cognitive deficits in PSDs.

## Highlights

• Entropy-based analysis of TVFC for brain-behavior mapping in early psychosis.
• Low-entropy patterns better encode performance on integrated cognitive tasks.
• High-entropy patterns better capture aspects of executive functioning.
• Entropy transitions align with oscillatory and neurotransmitter gradients.
• Low-entropy patterns relate to positive symptoms in early psychosis.

## 1 Introduction

Psychosis spectrum disorders (PSDs) are marked by positive and negative symptoms, as well as cognitive impairment (McTeague et al., 2017, Murray et al., 2004, Pearlson et al., 2016, Sharma et al., 2017). PSDs encompass traditionally distinct diagnostic categories like schizophrenia (SCZ) and bipolar disorder (Barch, 2017, Yamada et al., 2020), with positive symptoms like hallucinations and delusions being the predominant feature across these categories (van Os & Kapur, 2009). Positive symptoms have traditionally received a lot of attention in research on PSDs (Feinberg, 1978, Kapur, 2003, Sterzer et al., 2018), while cognitive dysfunction is less frequently discussed (Harvey et al., 2022). However, cognitive impairment is predictive of developing psychosis in high-risk individuals (Carrión et al., 2016, Seidman et al., 2016) and is a core feature across different manifestations of PSD (Bora & Pantelis, 2015). Consequently, mapping the neurophysiological correlates of cognitive performance in PSDs is an important subject of investigation, especially since the therapeutic effects of antipsychotic medications (APs) on cognitive deficits are merely moderate (Keefe et al., 2007), or even entirely absent for certain substances (Baldez et al., 2021). This mapping can be performed with functional magnetic resonance imaging (fMRI), where functional connectivity (FC) changes across large-scale brain networks in PSD patients have been reported (Anticevic et al., 2014, Cheng et al., 2015, Kambeitz et al., 2016, Ramsay, 2019, Woodward and Heckers, 2016). These changes are said to reflect dysfunctional integration of information between different brain systems with distinct roles in the processing hierarchy (Anticevic and Halassa, 2023, Friston et al., 2016), ultimately giving rise to the diverse set of PSD symptoms.

Besides FC, which is usually measured by the correlation between two blood-oxygen-level-dependent (BOLD) signals during rest, intrinsic properties of BOLD timeseries also reflect integrative processes (Garrett et al., 2018, Ito et al., 2020). The related concept of intrinsic neural timescales (INT) suggests that more self-similarity (longer INT) in a local BOLD signal reflects a longer temporal window for the integration of information within that brain region (Hasson et al., 2015, Stephens et al., 2013). Fittingly, INTs have been shown to be significantly shortened in PSD patients compared to healthy controls (Uscătescu et al., 2023, Uscătescu et al., 2021, Wengler et al., 2020), and follow a spatial gradient from primary-sensory to higher order regions (J. D. Murray et al., 2014, Raut et al., 2020). Relevant to the present investigation, the concept of INT can be extended to the level of connections (edges), by focusing on time-varying aspects of FC (TVFC). Although TVFC is still a controversial topic (Liegeois et al., 2017, Lurie et al., 2020), studying it has proven to be informative regarding interindividual differences (Liegeois et al., 2019, Vidaurre et al., 2021) and disease states (Jia et al., 2017, Kaiser et al., 2016, Sakoglu et al., 2010). Consequently, a small number of resting-state fMRI (rs-fMRI) studies have used sample entropy (SampEn) (Richman & Moorman, 2000) to quantify the self-similarity of edge fluctuations (edge-SampEn [ESE]) derived from sliding-window analysis (Hirsch and Wohlschlaeger, 2022, Jia and Gu, 2019b, Jia et al., 2017). SampEn is one way of assessing INT, with higher values corresponding to shorter INT (Omidvarnia et al., 2018, Sokunbi et al., 2014), and it is also significantly associated with mental abilities and cognitive load in healthy subjects (Menon and Krishnamurthy, 2019, Nezafati et al., 2020, Omidvarnia et al., 2022, Omidvarnia et al., 2021).

Evidence further indicates that ESE can provide complementary information to BOLD-derived SampEn (Menon & Krishnamurthy, 2019), suggesting potential use as a novel biomarker for neuropsychiatric conditions and their related symptoms. In support of this hypothesis, Jia and Gu (2019a) reported that ESE was significantly higher in SCZ patients at multiple spatial scales compared to healthy controls. However, statistical relationships between ESE and cognitive task-performance have not been explored in PSDs. Open questions also pertain to the possibly differential contributions of high and low ESE configurations to performance in patients: High ESE connections were most predictive of fluid intelligence in healthy subjects (Menon & Krishnamurthy, 2019). However, brain regions belonging to cortical networks associated with visuospatial and language functions display the lowest ESE in the brain (Hirsch & Wohlschlaeger, 2022), and their connectivity patterns have been repeatedly associated with cognitive ability (Hearne et al., 2016, Song et al., 2008, van den Heuvel et al., 2009). To address these issues, we analyze rs-fMRI and behavioral data from a clinical population (*n* = 97) of young adults that is within 5 years of onset of psychotic symptoms, as well as from healthy controls (*n* = 53). We contrast high and low ESE network configurations, in terms of their ability to explain behavioral variance across cognitive tasks in patients.

Given the evidence cited above, we hypothesize that their respective explanatory power would significantly depend on the specific cognitive task in question: Low ESE configurations should be more informative in tasks that need higher degrees of information integration. Conversely, high ESE configurations might better capture behavioral variance in tasks that depend more on ‘just’ the precise encoding of low-level stimulus features. Overall, we hope to generate new perspectives regarding the topography of neurophysiological correlates of cognitive performance in PSDs, through examining the timescales of TVFC with ESE. By combining our fMRI results with public data of neurotransmitter systems and brain oscillations (Hansen et al., 2022), we aim to gain more insight into the biological mechanisms underlying ESE configurations and their relationship with cognitive aspects of PSDs. This multimodal mapping of brain-behavior associations might help to generate new potential targets for therapeutic interventions in the cognitive domain, particularly since existing treatment options are only moderately effective (Vita et al., 2021).

## 2 Results

Imaging and behavioral data were taken from the Human Connectome Project for Early Psychosis (HCP-EP) open-source dataset (Section 4.1). Imaging data consisted of one rs-fMRI session (∼ 6 min) per subject (patients: *n* = 97; controls: *n* = 53). Behavioral data consisted of scores from the seven measures in the NIH-TB Cognition Battery (Weintraub et al., 2013), that capture individual variation across a range of cognitive subdomains. To map brain-behavior relationships in patients, we used multi- and univariate versions of a variance component model (Ge et al., 2016, Sabuncu et al., 2016), that has been recently employed to study TVFC–behavior associations in healthy subjects (Liegeois et al., 2019). In the context of our study, this linear mixed-effect model calculates how much of the variability in cognitive task performance among patients, both overall and for specific tasks, can be explained by variability in ESE patterns, while adjusting for covariates like age and medication (Section 4). After preprocessing, the functional data were parcellated into 116 regions (Section 4.1) and sliding-window analysis was conducted on the BOLD timeseries (Section 4.2). Given our window-size of 60 s, this resulted in 6670 correlational timeseries, each consisting of 316 temporally adjacent windows (Section 4.2). One SampEn value was then computed for each correlational timeseries, leading to a vector with 6670 elements for each subject (Section 4.2). In accordance with previous work, we then constructed high-entropy (HEN) and low-entropy (LEN) network templates, by selecting edges with the highest and lowest mean ESE values across healthy subjects (Hirsch & Wohlschlaeger, 2022). This was done for a range of different thresholds, and for every threshold we extracted the corresponding ESE values from the patients, which were then correlated (Pearson correlation) across patients to derive the similarity matrices to be put into the model (Ge et al., 2016). We then ran the behavioral model for each similarity matrix corresponding to a given threshold, and in the end selected the threshold that performed best for HEN and LEN (respectively) for the final analysis (Section 4.3). The two resulting (97×97) similarity matrices *RHEN* and *RLEN* (representing shared variance in ESE across patients) were then used as separate inputs for the variance component model to predict variance across and within cognitive domains. All ensuing behavioral analyses are based on comparing the outcomes from running the model separately for *RHEN* and *RLEN*.

Moreover, we performed basic topological analyses at the node-level, based on binarized versions of the (group-level) HEN and LEN templates, derived from the controls. This was done to replicate our previous finding that ESE is topographically organized along a subcortical (SC) to cortical axis in healthy subjects (Hirsch & Wohlschlaeger, 2022). Finally, to gain more insight into the neurobiological mechanisms underlying HEN and LEN configurations at the cortical level, we analyze their spatial correspondence with neurotransmitter maps derived from positron emission tomography (PET) and the topography of brain rhythms from magnetoencephalography (MEG). We use high-quality open-source data that combines the results from different studies (Hansen et al., 2022). We apply rigorous control for statistical dependencies between spatially adjacent brain regions through the employment of null-models matching the spatial autocorrelation of the empirical maps (Burt et al., 2020).

### 2.1 LEN encodes more information across cognitive domains in patients

**Fig. 1:**
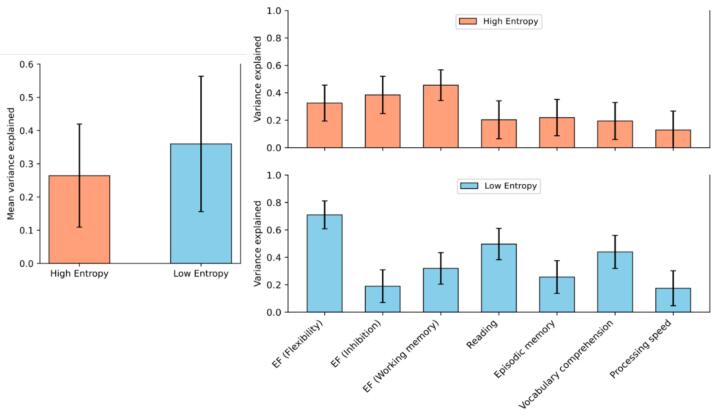
**Multivariate results.***Left:* Average variance explained across all cognitive measures is significantly lower for the High-entropy patterns (red), compared to the Low-entropy patterns (blue) in patients. Error bars represent parametric SEs. *Right:* Explained variance in patients, stratified by cognitive measure, for High-entropy (top row) and Low-entropy (bottom row). Error bars represent SEs derived from a bootstrapping procedure (see Appendix). EF = Executive functions; SE = Standard error. (For interpretation of the references to color in this figure legend, the reader is referred to the web version of this article.)

**Fig. 2:**
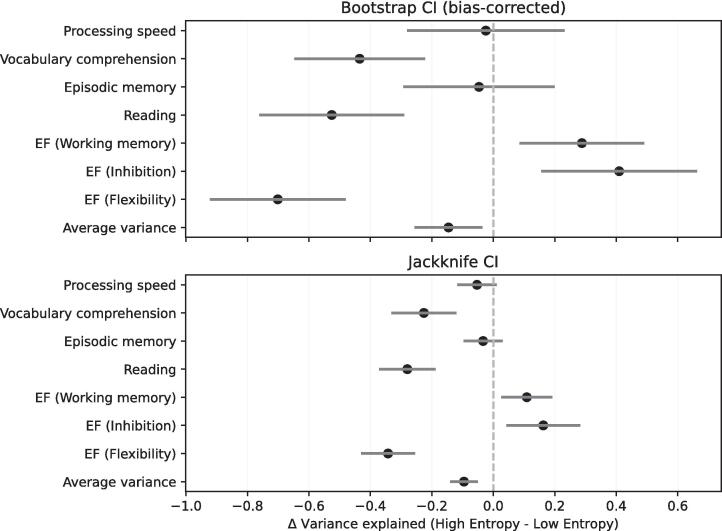
**Statistical inference.***Top:* Bias-corrected bootstrap CIs for the patients’ difference in variance explained (High-entropy vs. Low-entropy), stratified by cognitive measure. The dashed line denotes zero difference. *Bottom:* Confirmatory jackknife CIs (see Appendix) for the patients’ difference in variance explained (High-entropy vs. Low-entropy), stratified by cognitive measure. The dashed line denotes zero difference. CI = Confidence interval; EF = Executive functions.

On average, similarity in the HEN explained significantly less behavioral variance (26 %; *SE =* 16 %), compared to the LEN (36 %; *SE =* 20 %), bias-corrected bootstrap confidence-interval (BS-CI) of the difference [−26 %, −4%], Bonferroni corrected (Fig. 1, Fig. 2). There was reasonable evidence for the average explanatory variance to be significantly different from zero for both LEN (*p*-Wald = 0.0387, *p*-Perm = 0.034), and HEN (*p*-Wald = 0.0443, *p*-Perm = 0.045). Running the model after shuffling the original edges (independently for each patient) or selecting random edges (consistently across patients) resulted in higher *p*-values for both HEN (shuffled-edges: *p*-Wald = 0.4477, *p*-Perm = 0.4270; random-edges: *p*-Wald = 0.1602, *p*-Perm = 0.1560) and LEN (shuffled-edges: *p*-Wald = 0.4858, *p*-Perm = 0.4610; random-edges: *p*-Wald = 0.1665, *p*-Perm = 0.1650).

### 2.2 Explanatory power is domain specific

On the level of single measures, we observed significant interactions in subdomains of Executive function (EF): HEN (39 %; SE = 14 %) explained more variance in Inhibition (INH), compared to LEN (19 %; SE = 12 %), BS-CI [16 %, 66 %]. HEN (46 %; SE = 11 %) also explained significantly more variance in Working-memory (WM), compared to LEN (32 %; SE = 12 %), BS-CI [9 %, 49 %]. Conversely, HEN (33 %; SE = 13 %) explained significantly less variance in Flexibility, compared to LEN (71 %; SE = 10 %), BS-CI [-92 %, −48 %] (Fig. 2). Additionally, HEN was significantly less informative in Reading (20 %; SE = 14 %), compared to LEN (50 %; SE = 11 %), BS-CI [-76 %, −29 %]. Finally, HEN was also significantly less informative in Vocabulary comprehension (20 %; SE = 14 %), compared to LEN (44 %; SE = 12 %), BS-CI [−65 %, −22 %]. All BS-CIs were (Bonferroni) adjusted for multiple comparisons, and the results were insensitive to the choice of resampling method (Fig. 2).

### 2.3 Explanatory power is network specific

**Fig. 3:**
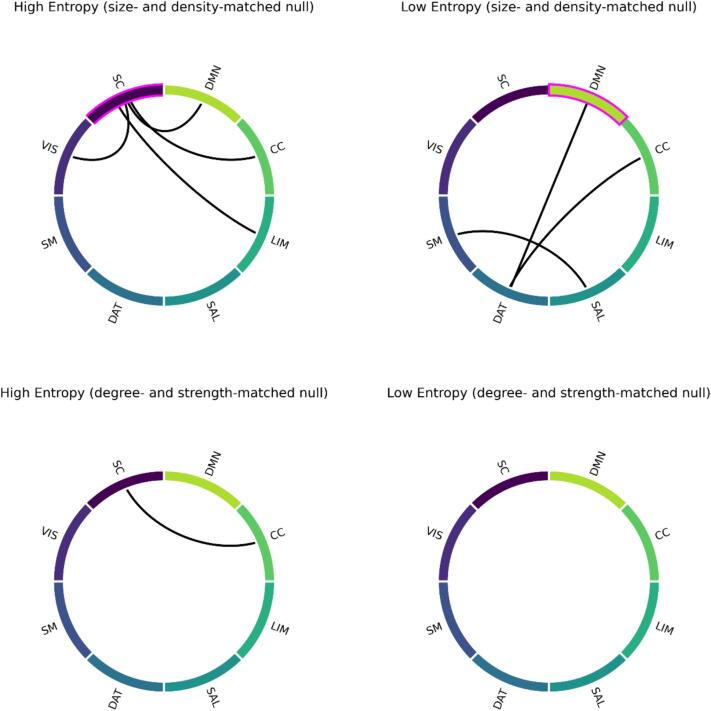
**Univariate results.***Top row:* Network interactions explaining significant behavioral variance across all cognitive measures in patients (see Appendix), for High-entropy (left) and Low-entropy (right). Significance was determined by randomly shuffling edges (10,000 permutations), before averaging within and between networks. Significant within-network explanatory variance is denoted by magenta colors. *Bottom row:* Network interactions explaining significant behavioral variance across all cognitive measures in patients, for High-entropy (left) and Low-entropy (right). Significance was determined by rewiring edges while matching the initial degree and strength distributions (10,000 permutations), before averaging within and between networks. CC = Cognitive Control; DAT = Dorsal Attention; DMN = Default-mode; LIM = Limbic; SAL = Salience; SC = Subcortical; SM = Somatomotor; VIS = Visual, see (Yeo et al., 2011). (For interpretation of the references to color in this figure legend, the reader is referred to the web version of this article.)

To evaluate behavioral variance explained for HEN and LEN at the level of networks, a univariate version of the multivariate variance component model was used, which resulted in an edgewise estimate quantifying the average amount of variance explained across all dependent variables (Fig. 3 and Appendix). We averaged edges-values within and between the boundaries of a SC, as well as seven established cortical resting-state networks (Yeo et al., 2011). These pertained to Visual- (VIS), Somatomotor- (SM), Dorsal Attention- (DAT), Salience/Ventral Attention- (SAL), Limbic- (LIM), Cognitive Control- (CC), as well as Default-mode (DMN) networks. To determine significance, we compared these empirical values against a series of values derived from 10,000 iterations of different null-models (see Appendix). We found that for the HEN, the average variance explained within SC was significantly higher than what would be expected based on a series of size- and density-matched random networks (*p*-Perm = 0.0014) (Fig. 3; top left). The same was true for SC interactions with VIS (*p*-Perm = 0.0014), LIM (*p*-Perm = 0.0014), CC (*p*-Perm = 0.0257), and DMN (*p*-Perm = 0.0014) (Fig. 3; top left). When compared to degree- and strength-matched random networks, only SC interactions with CC (*p*-Perm = 0.0072) remained significant (Fig. 3; bottom left). For the LEN, the average variance explained within DMN was significantly higher than what would be expected based on series of size- and density-matched random networks (*p*-Perm = 0.0261), as well as DMN interactions with DAT (*p*-Perm = 0.0456) (Fig. 3; top right). The same was true for SM interactions with SAL (*p*-Perm = 0.0014), and DAT interactions with CC (*p*-Perm = 0.0252) (Fig. 3; top right). When compared to degree- and strength-matched random networks, no interaction in the LEN explained significantly more behavioral variance across behavioral measures than expected (on average) (Fig. 3; bottom right). All reported *p*-values were controlled with FDR (Benjamini & Hochberg, 1995).

### 2.4 Spatial layout of ESE recapitulates SC-cortical axis in controls

**Fig. 4:**
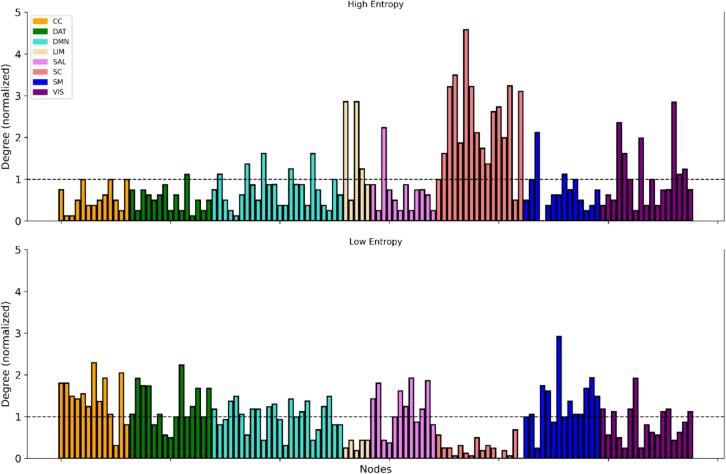
**Topological results for all nodes.***Top row:* Normalized degree-centrality of all nodes for High-entropy, stratified by network membership. Normalization was done via randomly shuffling edges (10,000 permutations), the dashed line denotes significance. *Bottom row:* Normalized degree-centrality of all nodes for Low-entropy, stratified by network membership. Normalization was done via randomly shuffling edges (10,000 permutations), the dashed line denotes significance. CC = Cognitive Control; DAT = Dorsal Attention; DMN = Default-mode; LIM = Limbic; SAL = Salience; SC = Subcortical; SM = Somatomotor; VIS = Visual, see (Yeo et al., 2011).

**Fig. 5:**
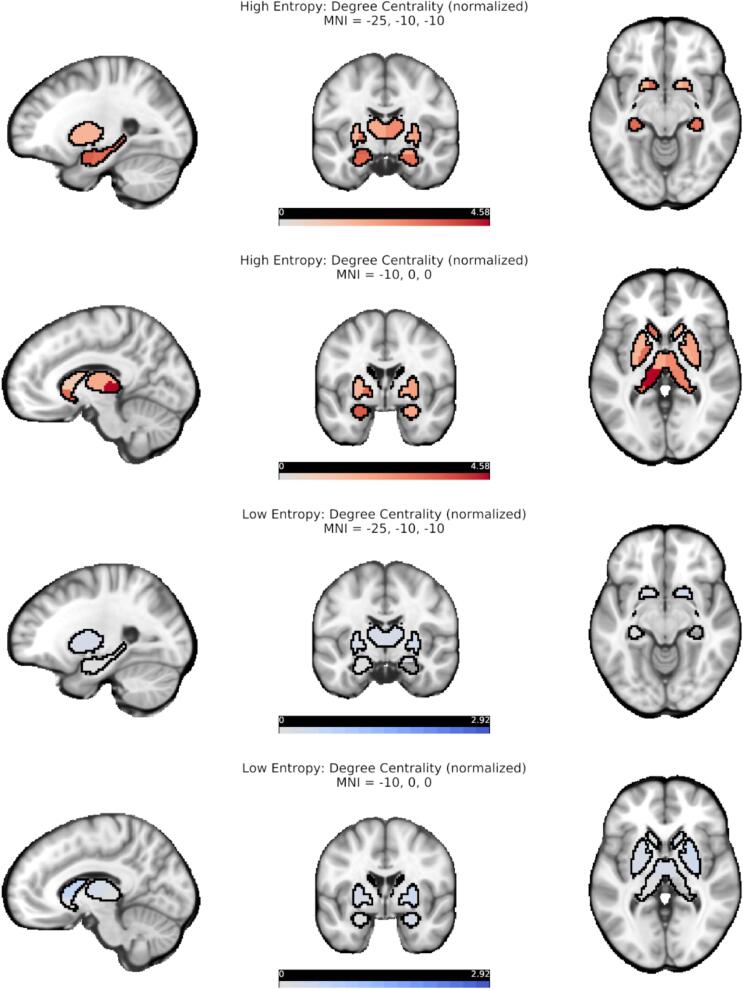
**Topological results for subcortical nodes.** Normalized degree-centrality of subcortical nodes for High-entropy (red) and Low-entropy (blue), depicted on representative slices of a structural image in MNI space. Normalization was done via randomly shuffling edges (10,000 permutations), darker colors denote higher degree-centrality. Data are the same as in Fig. 4. (For interpretation of the references to color in this figure legend, the reader is referred to the web version of this article.)

**Fig. 6:**
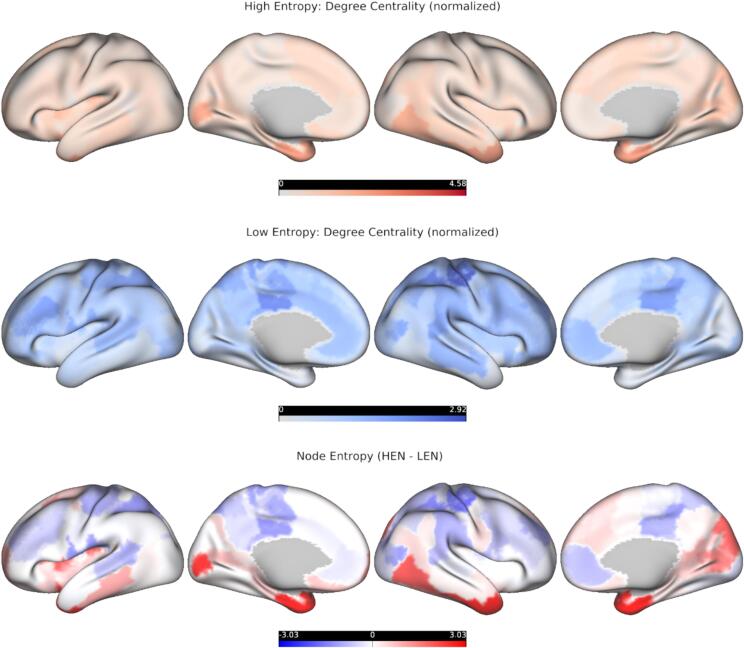
**Topological results for cortical nodes.***Top row:* Normalized degree-centrality of cortical nodes for High-entropy (red colormap), projected onto an inflated representation of the cortical surface. Darker colors denote higher normalized degree. Normalization was done via randomly shuffling edges (10,000 permutations). Data are the same as in Fig. 4. *Middle row:* Normalized degree-centrality of cortical nodes for Low-Entropy (blue colormap), projected onto an inflated representation of the cortical surface. Darker colors denote higher normalized degree. Normalization was done via randomly shuffling edges (10,000 permutations). Data are the same as in Fig. 4. *Bottom row:* Cortical Node-Entropy, obtained by subtracting the normalized-degree centralities (High-entropy – Low-entropy), projected onto an inflated representation of the cortical surface and z-scored for visualization purposes. Darker red colors denote higher Node-Entropy, darker blue colors lower Node-Entropy. Data are the same as in Fig. 4. (For interpretation of the references to color in this figure legend, the reader is referred to the web version of this article.)

We assessed the relative importance of single regions to HEN and LEN configurations by computing the (binary) degree-centrality for each node in the respective templates (Section 2). Each node’s degree-centrality was normalized by the mean degree-centrality from a series of size- and density-matched random networks (Fig. 4). For the HEN, the highest values were localized in SC, with left hemispheric nodes in the posterior thalamus, amygdala, and hippocampus at the top (Fig. 4, Fig. 5). Cortical nodes with the highest values were found in LIM regions of the temporal lobes (bilaterally), as well as in areas belonging to VIS (Fig. 4, Fig. 5, Fig. 6). For the LEN, highest values belonged to pre- and postcentral SM and DAT regions (bilaterally), as well as to bilateral prefrontal- and cingulum areas of the CC (Fig. 4 and Fig. 6). Overall, the degree-centralities were spatially organized in strong correspondence with our previous results in large sample of young and healthy subjects (Hirsch & Wohlschlaeger, 2022).

### 2.5 Topography of ESE mirrors macroscale patterns of cortical organization

**Fig. 7:**
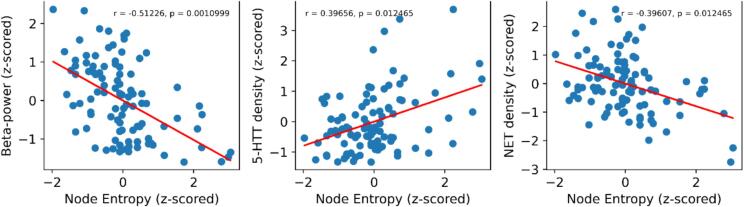
**Node-Entropy and macroscale patterns of cortical organization.** Scatterplots depicting the relationship between Node-Entropy (x-axis) and beta-power (y-axis; left), 5-HTT density (y-axis; middle), and NET density (y-axis; right) for cortical nodes. Red lines denote least-square fits from linear regression. All variables were z-scored for visualization. *P*-values were derived with spatial-surrogate testing and then controlled with FDR (Section 2.5). 5-HTT = Serotonin-transporter; NET = Norepinephrine-transporter. (For interpretation of the references to color in this figure legend, the reader is referred to the web version of this article.)

**Fig. 8:**
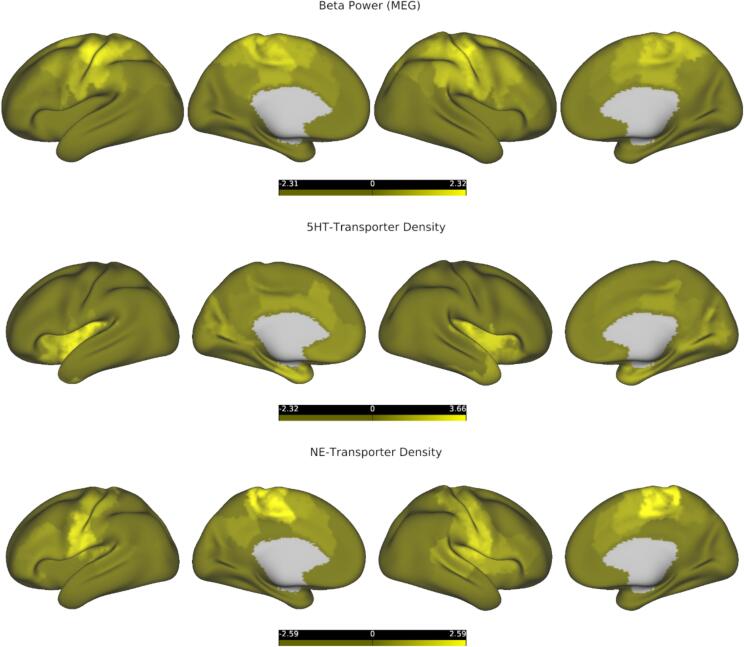
**Macroscale gradients of cortical organization.***Top row:* MEG beta-power, projected onto an inflated representation of the cortical surface and z-scored for visualization purposes, lighter colors denote higher beta power. *Middle row:* 5-HTT-density, projected onto an inflated representation of the cortical surface and z-scored for visualization purposes, lighter colors denote higher density. *Bottom row:* NET-density, projected onto an inflated representation of the cortical surface and z-scored for visualization purposes, lighter colors denote higher density. Data were taken from a public repository (Section 4.1). 5-HTT = Serotonin-transporter; MEG = Magnetoencephalography; NET = Norepinephrine-transporter.

We combined the normalized HEN/LEN degree-centrality estimates for each cortical node by subtracting them from each other (HEN – LEN) before rescaling them to the interval [0, 1] (Fig. 6, bottom row). The resulting Node-Entropy value captures a region’s trend towards being central in either HEN or LEN, at the behaviorally most informative density of these respective configurations (Section 2). These values were then correlated with the corresponding values of a series of spatial maps denoting densities of different neurotransmitter receptors/transporters (from PET), and oscillatory-power within predefined frequency bands (from MEG), see Hansen et al. (2022) for details (Fig. 8). We restricted our analyses to maps for which the absolute Pearson correlation with Node-Entropy was at least 0.2. For each map significance was determined by comparing the empirical correlation value to a corresponding distribution derived from 10,000 surrogate maps preserving the spatial autocorrelation of the initial map (Burt et al., 2020), and finally these *p*-values were controlled with FDR. We found that Node-Entropy was significantly anticorrelated with MEG beta-power (*r* = −0.51, *p* = 0.001) and density for the norepinephrine transporter (NET; *r* = −0.4, *p* = 0.012) (Fig. 7). It was also significantly correlated with density for the serotonin transporter (5-HTT; *r* = 0.4, *p* = 0.012) (Fig. 7). These results indicate that ESE at the node level tracks spatial gradients related to large-scale neuronal dynamics and neurotransmission. Importantly, they also offer valuable additional information to properly interpret the relationships between ESE and different cognitive domains in PSD we have described above.

### 2.6 Additional analyses

#### 2.6.1 LEN maps integration

**Fig. 9:**
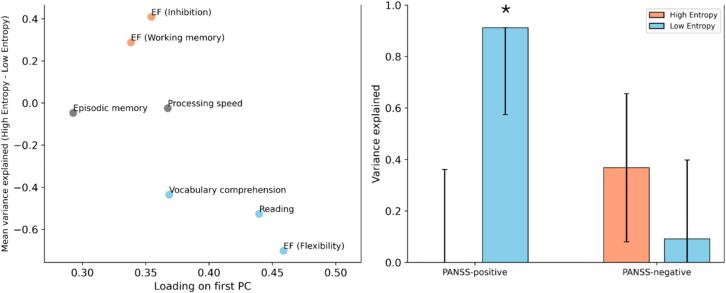
**Additional multivariate (left) and exploratory univariate results (right).***Left:* Scatterplot visualizing the relationship between integration (loading on the first PC; x-axis) and difference in variance explained (High-entropy vs. Low-entropy; y-axis), stratified by cognitive measure. Measures are color-coded according to direction and significance of the difference (blue: High-entropy < Low-entropy; red: High-entropy > Low-entropy; grey: non-significant). *Right:* Low-entropy (blue) significantly explains variance in positive symptom severity in patients (PANSS-positive). Error bars denote parametric SEs and significance is indicated by an asterisk. PANSS = Positive and Negative Syndrome Scale; PC = Principal Component; SE = Standard error. (For interpretation of the references to color in this figure legend, the reader is referred to the web version of this article.)

Our pattern of results is compatible with the hypothesis that LEN configurations preferentially encode behavioral information on tasks that require extensive information-integration (such as language and knowledge-based tasks), while HEN tends to explain more variance on tasks geared more towards quick and reliable extraction of stimulus features (EF tasks measuring INH and WM). However, this a-priori grouping into cognitive domains (language vs. EF) is problematic and not clearly reflected in our findings, given that LEN explains the most variance in the EF ‘subdomain’ of Flexibility (Fig. 1, Fig. 2). To further test our hypothesis in a data-driven way, we conducted a principal component analysis on the cognitive variables from the whole sample. The first principal component, which explained approximately 52 % of the variance, had positive loadings from all cognitive variables, indicating that it represented shared features across all tasks and their related domains (Fig. 9, left). Interestingly, the average loading on the first PC was significantly lower for variables whose variance was significantly better explained by HEN (INH, WM), compared to variables that were more related to LEN (Flexibility, Reading, and Vocabulary comprehension), 95 % BS-CI [-0.14; −0.02] (Fig. 9, left). It seems that in terms of explanatory power, LEN configurations outperform HEN configurations specifically on tasks that engage a wide range of cognitive domains, possibly accompanied by higher degrees of integrative and distributed processing on a neuronal level.

#### 2.6.2 LEN relates to positive PSD pathology

While our main aims in this study were connected to cognitive variables, we also performed explorative analyses to see if and how HEN and LEN signatures would relate to the positive and negative symptom complexes that characterize PSD pathology. To do this we ran the univariate version of the statistical model (see Appendix) with the patients scores on the Positive and Negative Syndrome Scale (PANSS) (Liechti et al., 2017) as dependent variables (separately). The same covariates were used as in the analyses of the cognitive data (Section 4.4). We found that LEN significantly encoded inter-patient variance on the positive scale (91 %; *SE =* 34 %; *p*-Wald = 0.0034), which was not the case for HEN (Fig. 9, right). Additionally, there was some weaker evidence that HEN significantly explained variance on the negative scale (37 %; *SE =* 29 %; *p*-Wald = 0.11) (Fig. 9, right). Although these results from the univariate model should be interpreted with caution (Sabuncu et al., 2016), they show a correspondence between our suggested marker of neuronal integration (ESE) and core PSD symptoms like hallucinations and delusions, which fittingly have been hypothesized to stem from faulty integration of information between sensory and higher order brain systems (Anticevic and Halassa, 2023, Friston et al., 2016).

## 3 Discussion

### 3.1 Summary

In this study, we show that the timescales of TVFC during rest significantly encode information about cognitive task performance in a large sample of young adults in the early phases of psychosis. Our hypothesis was that diverging levels of ESE in patients would be differentially related to cognitive variables, depending on the level of integrative processing required for performing the related task. We find that brain configurations marked by low ESE (high integration; LEN) explain significantly more behavioral variance overall in patients, compared to constellations designated by high ESE (low integration; HEN). In line with our proposal, this result is driven by LEN encoding significantly more variance on tasks that engage a wider range of cognitive processes (Section 2.6.1). Fittingly, the most informative connections of the LEN are distributed across a range of cortical regions, encompassing unimodal as well as higher-order cortical networks (Section 2.3 and Fig. 3, Fig. 4). In contrast, the most informative HEN connections are concentrated between SC and CC (Fig. 3, bottom left). This mostly reflects significantly higher relative explanatory power for HEN in tasks related to (empirically) more isolated EF subdomains (WM, INH). In sum, ESE is a useful marker for disentangling the relative contributions of different brain systems to specific aspects of cognitive performance in PSD.

In healthy controls, ESE decreases along a SC to cortical gradient and is lowest for nodes belonging to SM, DAT and CC (Fig. 4, Fig. 5, Fig. 6), which replicates our previous findings (Hirsch & Wohlschlaeger, 2022). For cortical areas, Node-Entropy closely covaries with oscillatory power in the beta frequency-range during rest (ca. 12–30 Hz), with lower values corresponding to higher beta-power (Fig. 7, Fig. 8). This points towards a possible neurobiological mechanism through which information could be integrated in the LEN, especially since Node-Entropy also tracks the density of NET (Fig. 7, Fig. 8), with lower values corresponding to higher density. Additionally, Node-Entropy is significantly related to 5-HTT density, with higher values corresponding to higher density (Fig. 7, Fig. 8). Overall, these results provide valuable insights into the oscillatory and neuromodulatory profiles of HEN and LEN. Given that the edge dynamics of PSD patients within these configurations are significantly related to their cognitive profiles, they also provide a mechanistic framework for possible interventions to improve cognition in PSDs. This is especially important, since our explorative analyses also show that LEN dynamics in patients are significantly related to positive symptoms like hallucinations and delusions (Fig. 9, right). In the following sections we will discuss the implications of our findings in the context of the existing literature.

### 3.2 Relationships with previous work

The outcomes of the present investigation validate the substantial body of literature showing that resting-state dynamics are useful biomarkers for neuropathological conditions (Bauer et al., 2022, Kaiser et al., 2016, Rashid et al., 2016, Ries et al., 2019, Sakoglu et al., 2010, Salman et al., 2019, Zöller et al., 2019), and that ESE significantly relates to behavior and cognition (Jia and Gu, 2019a, Jia et al., 2017, Menon and Krishnamurthy, 2019). However, our detailed mapping of HEN/LEN configurations and their multimodal profiles to specific aspects of cognition and positive pathology in PSDs provides new theoretical insights and has possible clinical utility.

#### 3.2.1 LEN

Networks significantly related to cognitive performance in the LEN include DMN, SAL and CC, all of whom are part of the triple-network model of general psychopathology (V. Menon, 2011). The model postulates that cognitive deficits in SCZ and psychopathology in general arise from dysfunctional interactions between these higher order regions (Menon and Uddin, 2010, Palaniyappan and Liddle, 2012). Indeed, their dysfunction is predictive of cognitive deficits across modalities and diagnostic criteria (Sheffield et al., 2017, Sui et al., 2018), which also holds true in the present investigation. Since the LEN/HEN templates were derived from group-average ESE values across healthy individuals (Section 2), our results partially reflect the spatial gradient of ESE (Fig. 4, Fig. 5, Fig. 6), with nodes belonging to these higher order regions amid the most highly connected in the LEN. However, the behavioral significance of the patients LEN dynamics was entirely absent when edges were selected randomly (Section 2.1). This suggests that the TVFC timescales within and between those areas were indeed amongst the most informative about specific aspects of cognition. The lack of significance for any within- and between-network interaction after controlling for degree and strength (Fig. 3, bottom right) indicates that the behavioral relevance of LEN is not so much concentrated but rather distributed across its constituent nodes and associated systems. This conceptually aligns with our finding that LEN dynamics preferentially encode performance in cognitive tasks requiring higher degrees of integration.

The fact that the topography of Node-Entropy was strongly anticorrelated with MEG beta-power (Fig. 7) implies coordinated activity between nodes within the LEN, underscoring that this configuration is not merely incidental. Beta-power has been related to ongoing effortful cognition (Schmidt et al., 2019) and motor-preparation/execution (Baker, 2007, Pfurtscheller and Berghold, 1989, Tewarie et al., 2018). Interestingly, this rhythm seems to be important for integrating bottom-up and top-down signals (Tan et al., 2016), and tracks SCZ pathology (Donati et al., 2021, Gascoyne et al., 2021, Pittman-Polletta et al., 2015). LEN dynamics were also significantly related to positive symptom severity in the present study (Fig. 9, right), providing further evidence for a possible link between ESE and large-scale neuronal dynamics. The Node-Entropy connection to oscillatory behavior should be interpreted together with the corresponding spatial correlations with NET- and 5-HTT densities (Fig. 7). Recent evidence shows that these distributions significantly predict the topography of MEG-derived beta-power (Hansen et al., 2022), and monoaminergic dysfunction is central to many explanatory accounts of PSDs (Davis et al., 1991, Eggers, 2013). The noradrenergic system has been implicated in the cognitive deficits of SCZ patients (Mäki-Marttunen et al., 2020), and has been hypothesized to drive integration between distributed brain systems through neural gain (Shine, 2019, Totah et al., 2018). Since Node-Entropy was significantly anticorrelated with NET density, these accounts align with our notion that ESE inversely tracks integration. Behaviorally this is reflected in the (relatively) superior performance of patients’ LEN patterns to encode variance in psychometrically more integrated tasks, which evidence suggest require higher degrees of distributed processing (Colom et al., 2006, Dajani and Uddin, 2015, Niendam et al., 2012).

A corollary of our results is that psychoactive interventions that target positive symptoms in PSDs should also significantly influence cognitive performance, given that LEN dynamics were significantly related to both aspects of the pathology. There is indeed evidence that some APs have small positive effects on cognition (Baldez et al., 2021, Davidson et al., 2009), with negative effects also being reported (Sakurai et al., 2013). Of note, a recent network meta analysis showed that the APs haloperidol and clozapine, which are known for their antagonistic effects on noradrenergic transmission, had the most detrimental effects on global cognition (Baldez et al., 2021). This is compatible with our present results that show a noradrenergic involvement in the LEN dynamics, which significantly encode cognitive-task variance in PSD patients. Interestingly, NET can also modulate dopaminergic signaling, especially in CC related areas (Gresch et al., 1995, Mäki-Marttunen et al., 2020, Morón et al., 2002), and dopamine dysfunction has been the central element in many theories of PSDs (Howes & Kapur, 2009).

#### 3.2.2 HEN

Areas significantly related to behavior in the HEN pertained to interactions within SC as well as SC interactions with VIS and higher order networks (Fig. 3, top/bottom left). These associations were driven by HEN explanatory power in specific tasks (List sorting and Flanker) related to EF subdomains (WM and INH). These tasks require quick and precise encoding of low-levels stimulus features to perform well (Tulsky et al., 2013, Zelazo et al., 2013). Our results suggest that connections with high ESE (low integration) best encoded this ability during rest in PSD patients, which is compatible with our hypothesis. This is in line with evidence that SC and VIS areas have shorter INTs, compared to (cortical) higher order areas (Muller et al., 2020, Raut et al., 2020), which is also true for the timescales of TVFC (Hirsch & Wohlschlaeger, 2022). Interactions within SC are proposed to act as shortcuts for rapid sensory processing (McFadyen et al., 2020), and SC-cortical interactions have been consistently associated with cognitive symptoms in PSDs (Anticevic and Halassa, 2023, Peters et al., 2016, Ramsay, 2019), possibly also influencing cortico-cortical connectivity (Hirsch & Wohlschlaeger, 2023). The behaviorally most informative HEN interactions were between SC and CC (Fig. 3, bottom left), contrasting the more distributed nature of relevant LEN edges. In general, ESE was able to dissociate different aspects of EF (WM/INH vs. Flexibility) in terms of their neurophysiological correlates, in line with the proposed *stability* vs. *flexibility* dichotomy of cognitive control (Fuster, 2015, Sakai, 2008).

The observed significant correlation between 5-HTT density and Node-Entropy for cortical nodes indicates an involvement of the serotonergic system in HEN dynamics (Fig. 7), especially since FC changes after 5-HTT blockage have been reported for the central HEN regions including the thalamus, amygdala, and VIS (Boucherie et al., 2023). Moreover, serotonergic signaling under normal conditions has been related to (SC driven) feedforward cortical processing (Shine et al., 2022), which is associated with shorter timescales (Bastos et al., 2012). Of note, performance in WM and selective attention (akin to INH) was improved for PSD patients after administration of the AP olanzapine, relative to other atypical APs, typical APs, and placebo (Baldez et al., 2021, Woodward et al., 2005). These improvements were partially attributed to olanzapine’s increased affinity for some serotonergic receptors (Baldez et al., 2021, Bymaster et al., 2001, Woodward et al., 2005), aligning with evidence showing serotonergic effects on WM (Williams et al., 2002) and INH (Pattij & Schoffelmeer, 2015). Collectively, these findings suggest a neurobiological basis for our observed relationship between HEN timescales and specific aspects of cognition in PSD.

### 3.3 Limitations

While our hypothesis was based on the notion from INTs that more self-similarity indicates a greater potential for integration (Hasson et al., 2015; J. D. Murray et al., 2014), ESE is only indirectly related to the BOLD signal via TVFC. However, our findings in PSD patients indeed suggest that TVFC configurations marked by more regular fluctuations (low ESE) explain more variance on tasks that require more integrated processing (Fig. 9). TVFC fluctuations have been interpreted as shifting brain-states (Allen et al., 2014, Leonardi and Van De Ville, 2015), reflecting underlying electrophysiological phenomena (Tagliazucchi et al., 2012, Thompson, 2018) and neuromodulatory processes (Shafiei et al., 2019, Shine, 2019), which is compatible with our findings. A criticism of our methodology could be that ESE might not be sensitive to active communication between two given regions. It is certainly possible for an edge to have low ESE (high integration) but for the two corresponding nodes to have low or negative FC. However, we do not think that such connections should be excluded or that their existence invalidates our interpretation of ESE. On the contrary, evidence shows that weak connections are especially informative about cognition (Santarnecchi et al., 2014) and topological changes in PSDs (Bassett et al., 2012, Mastrandrea et al., 2021).

Another possible issue is that SampEn (by definition) is influenced by basic signal properties like temporal signal-to-noise ratio (Keilholz et al., 2020), which is lower for BOLD signals from SC and temporal regions. Although we have shown in the past that the implications for ESE are small (Hirsch & Wohlschlaeger, 2022), these influences must be kept in mind when interpreting spatial patterns of ESE. Consequently, we correlated the degree-centrality maps (Node-Entropy, HEN, and LEN) with a temporal signal-to-noise ratio map (averaged across healthy subjects) as a control analysis. None of the correlations were significant, with all *p* > 0.05 (uncorrected): Node-Entropy (*r* = 0.15, *p* = 0.16), HEN(*r* = 0.17, *p* = 0.08), and LEN (*r* = −0.1, *p* = 0.72). On a different note, we and others have equated high (single-scale) SampEn with high complexity in the past (Hirsch and Wohlschlaeger, 2022, Jia and Gu, 2019a, Jia and Gu, 2019b, Jia et al., 2017), but some have argued that such an interpretation requires a multi-scale entropy analysis (Costa et al., 2002, Yang et al., 2015). While we have avoided the notion of complexity in the present study, it should be noted that contrary to BOLD SampEn, ESE at our scale of interest captured most of the behaviorally relevant information in healthy subjects (Menon & Krishnamurthy, 2019). Finally, our results pertain to effects across the psychosis spectrum and not directly to more narrowly defined diagnostic categories like SCZ and bipolar disorder, making such analyses an interesting prospect for future cross-sectional and longitudinal investigations.

### 3.4 Clinical implications

The main (potential) clinical utility of our findings lies in the association between distinct aspects of cognition in PSDs and the topography of neurotransmitter and oscillatory systems, via ESE. Although (small) positive effects of APs on cognition have consistently been reported (Baldez et al., 2021, Keefe et al., 2007), our findings suggest that pharmacological interventions specifically aimed at noradrenergic and/or serotonergic systems (such as selective serotonin reuptake inhibitors [SSRIs]) might prove beneficial in terms of improving specific aspects of cognition in PSDs and related disorders. Along these lines, some positive (cognitive) effects of SSRIs have been reported in PSDs (Mancini et al., 2021, Silver et al., 2015), but no clinically relevant effects of SSRIs and noradrenergic anti-depressants on cognition were found in a recent metanalysis of chronic SCZ patients (Vernon et al., 2014). However, the included studies were small, and cognitive outcomes were grouped within a-priori cognitive domains (EF, language, etc.) (Vernon et al., 2014). Our results suggest that this grouping could obscure possible positive effects. In addition, our findings pertain to young patients across the PSD spectrum, not chronic SCZ. Finally, the implication of the beta-rhythm in the LEN makes it a potential target for brain-stimulation techniques, which is technically feasible with non-invasive methods (Hannah et al., 2022).

## 4 Materials and Methods

### 4.1 Sample characteristics and image preprocessing

The initial sample consisted of the 169 subjects for which minimally preprocessed structural data was available at the time of download as part of the HCP-EP 1.1 Release (https://www.humanconnectome.org/study/human-connectome-project-for-early-psychosis/document/hcp-ep). For these subjects the (volumetric) minimal preprocessing pipeline of the Human Connectome Project (HCP) was conducted, see (Glasser et al., 2013, Smith et al., 2013) for details. Briefly, one rs-fMRI run lasted 5 min and 47 s, 2 mm isotropic resolution, multiband acceleration factor 8, TR = 0.8 s, TE = 0.037 s, phase encoding direction posterior-to-anterior. Additional runs were available in the anterior-to-posterior direction, but we only used one run in the posterior-to-anterior direction per subject, to ensure better signal accuracy in frontal regions. Preprocessing delivered unsatisfying results for five subjects due to issues with the field-maps, which were subsequently excluded from further analysis. Out of the remaining 164 subjects, 150 subjects had sufficient behavioral data available (patients: *n* = 97; controls: *n* = 53), which were then included in the final sample. Functional data were then denoised with aCompCor (Behzadi, Restom, Liau, & Liu, 2007), which included regressing out signals from white-matter regions and the ventricles (Muschelli et al., 2014). Additionally, the six movement parameters and their derivatives were regressed out, and the images were downsampled to 116 cortical and SC regions (Tian, Margulies, Breakspear, & Zalesky, 2020), with a template from (https://github.com/yetianmed/subcortex/blob/master/Group-Parcellation/3T/Cortex-Subcortex/MNIvolumetric/Schaefer2018_100Parcels_7Networks_order_Tian_Subcortex_S1_MNI152NLin6Asym_2mm.nii.gz). Preprocessed PET (*n* = 19) and MEG (*n* = 6) spatial maps were obtained from a public repository (https://github.com/netneurolab/hansen_receptors) at the 100 parcel resolution of the Schaefer atlas (Schaefer et al., 2018). Briefly, the downloaded PET images corresponding to different receptor/transporter densities were initially created by taking (weighted) averages across normalized maps from different (primary) studies using the same PET tracers, for details see (Hansen et al., 2022). MEG maps were initially derived from (young adult) HCP data (Van Essen et al., 2013) by (Shafiei, Baillet, & Misic, 2022).

### 4.2 Sliding-window and entropy calculations

Prior to sliding-window analysis, data were bandpass filtered from 0.017–0.1 Hz (Leonardi & Van De Ville, 2015), and the mean signal across all regions was regressed from the data (a version of global-signal regression). Global-signal regression has been shown to be beneficial for alleviating the influence of global artifacts in rs-fMRI data (Burgess et al., 2016), strengthen brain-behavior relationships on task measures (Li et al., 2019), and increases sensitivity to FC differences between controls and clinical populations (Parkes, Fulcher, Yücel, & Fornito, 2018). The first and last 10 frames were removed to account for any boundary effects. We used a rectangular window with a width corresponding to 60 s, which was then slid in steps of one TR across the timeseries. Within each window we computed the Pearson correlation between all regions, which was then Fisher-transformed prior to further analysis. Then SampEn was calculated for each correlational timeseries. To compute the SampEn for a given signal *x* = [$x_{1},\;x_{2},\;...\;,\;x_{N}$] with length *N*, an embedding vector with *m* running data points is derived from *x*: $v_{i}=\left[x_{i},\;x_{i+1},\;...\;,\;x_{i+m-1}\right]$, with *m* corresponding to the embedding dimension. For each *i* (1 *≤ i ≤ N* – *m*) define

$$C_{i}^{m}=\frac{1}{N-m-1}\sum_{j=1,\;j\neq i}^{N-m}\Theta \left(r-{\left\|v_{i}-v_{j}\right\|}_{1}\right),$$

where *r* = ε*σ*_*x*_ corresponds to a tolerance value, ε to a scaling parameter, and *σ*_*x*_ to the standard deviation of *x*. Θ(·) is the Heaviside function

$$\Theta \left(x\right)=\left\{\begin{array}{c} 0,x<0\\ 1,x\geq 0 \end{array}\right),$$

and ${\left\|\cdot \right\|}_{1}$ is the Chebyshev distance

$${∥v_{i}-v_{j}∥}_{1}=\max\left(|x_{i}-x_{j}|,|x_{i+1}-x_{j+1}|,\;\ldots \;,|x_{i+m-1}-x_{j+m-1}|\right).$$

Then, for each *i* (1 *≤ i ≤ N – m*) define

$$C_{i}^{m+1}=\frac{1}{N-m-1}\sum_{j=1,\;j\neq i}^{N-m}\Theta \left(r-{\left\|v_{i}-v_{j}\right\|}_{1}\right).$$

Averaging over all embedding vectors gives

$$U^{m}=\frac{1}{N-m}\sum_{i=1}^{N-m}C_{i}^{m},$$

and

$$U^{m+1}=\frac{1}{N-m}\sum_{i=1}^{N-m}C_{i}^{m+1}.$$

SampEn is then defined as

$$-\ln\left(U^{m+1}/U^{m}\right),$$

resulting in a nonnegative number, with higher values indicative of less regularity in the signal (Richman & Moorman, 2000). To ensure comparability of our results with past investigations, we used the standard parameter values of *m* = 2 and ε = 0.20 (Hirsch and Wohlschlaeger, 2022, Jia and Gu, 2019b). For BOLD signals of at least 97 timepoints evidence suggests that results from SampEn analyses are robust to parameter changes (Yang et al., 2018), and similar results were obtained for ESE (Jia et al., 2017).

### 4.3 Construction of HEN/LEN templates and similarity matrices

After ESE values were obtained for all subjects, HEN and LEN templates were constructed by proportional thresholding of the ESE matrix averaged across healthy individuals. For each cutoff only a certain proportion of the highest (HEN) or lowest (LEN) edges was kept. The resulting 32 templates (16 HEN and 16 LEN) were then used as binary masks to extract the corresponding ESE values from the patients, which were then correlated across patients to obtain the similarity matrices. We then ran the statistical model (Section 4.5) for each cutoff with the corresponding similarity matrices as inputs. Each cutoff was ranked according to explanatory power (mean variance explained), significance (*p*-Perm and *p*-Wald), and concordance (absolute difference between *p*-Perm and *p*-Wald). Briefly, a close correspondence between parametric and nonparametric *p*-values indicates that model assumptions are well met (Ge et al., 2016). Subsequently, the average rank across all criteria was calculated and the cutoff with the highest rank was chosen for all downstream analyses. For HEN the optimal cutoff was ∼ 6 % density and for LEN ∼ 14 % density. Importantly, the complexity of the models for HEN and LEN is equivalent, since the final model inputs (the similarity matrices *RHEN* and *RLEN*) have equal dimensions, see Liegeois et al. (2019) for a discussion.

### 4.4 Behavioral variables and covariates

The seven selected behavioral variables constitute the cognitive module of the NIH Toolbox for the Assessment of Neurological and Behavioral Function, which measures the cognitive domains of EF, episodic memory, language, processing speed, WM, and attention (Weintraub et al., 2013). Under EF we grouped the subdomains of Flexibility (Dimensional Change Card Sort), INH (flanker task), and WM (list sorting working memory test) (Tulsky et al., 2013, Zelazo et al., 2013). Language functions were denoted by Reading (Oral Reading Recognition Test) and Vocabulary comprehension (Picture Vocabulary Test) (Gershon et al., 2013), and Episodic memory was assessed with the Picture Sequence Memory Test (Bauer et al., 2013). Finally, Processing speed was assessed with the Pattern Comparison Processing Speed Test (Carlozzi et al., 2013). For all tests the age-corrected scaled scores were utilized (Weintraub et al., 2013). One subject had a missing score for Episodic memory, which was set to the median value across subjects. Prior to being entered into the model, the variables were quantile normalized to a Gaussian distribution to fit model assumptions (Liegeois et al., 2019). We included age (*M* = 22.65 years, *SD* = 3.36 years), sex (37 % female), current dose of AP medication (Chlorpromazine equivalents [*M* = 175.52 mg, *SD* = 234.29 mg]), mean framewise-displacement (*M* = 0.12 mm, *SD* = 0.06 mm) (Power et al., 2012), as well as phenotype-description (non-affective [*n* = 73] vs. affective psychosis [*n* = 24]) as covariates in the model. Briefly, non-affective psychosis participants met DSM-5 criteria for SCZ, schizophreniform, schizoaffective, psychosis NOS, delusional disorder, or brief psychotic disorder with onset within the past five years prior to study entry. Affective psychosis participants met DSM-5 diagnosis of major depression with psychosis (single and recurrent episodes) or bipolar disorder with psychosis (including most recent episode depressed and manic types) with onset within five years prior to study entry (https://www.humanconnectome.org/storage/app/media/documentation/data_release/HCP-EP_Release_1.0_Manual.pdf). One subject had a missing current-medication value, which was set to zero, given that the subjects lifetime exposure to APs was denoted by zero.

### 4.5 Variance component model

The multidimensional variance component model of Ge et al. (2016) takes the following form:

Y=C+E,

where Y, C, and E are 97×7 matrices, with Y representing the (quantile normalized) cognitive variables for all included *N* patients (Section 4.4). $\mathrm{Vec}\left(C\right)\;∼\;N\left(0,{\Sigma_{c}}_{⊗}R\right)$, and $\mathrm{Vec}\left(E\right)\;∼\;N\left(0,\Sigma_{e}⊗I\right)$, where $\mathrm{Vec}\left(.\right)$ is the vectorization operator, ⊗ the Kronecker matrix product, R the similarity matrix (i.e., either *RHEN* or *RLEN*) and I the identity matrix. The 7×7 matrices $\Sigma_{c}$ and $\Sigma_{e}$ are to be estimated from R and Y, which can be done with a moment-matching method (Ge et al., 2016):

$$\hat{\Sigma_{c}}=\frac{1}{\nu_{R}}Y^{T}\left(R-\tau I\right)Y,\;\;and\;\;\hat{\Sigma_{e}}=\frac{1}{\nu_{R}}Y^{T}\left(\kappa I-\tau R\right)Y,$$

where $\tau =\frac{\text{Tr}\left(R\right)}{N},\kappa =\frac{\text{Tr}\left(R^{2}\right)}{N},$ and $\nu_{R}=N\left(\kappa -\tau^{2}\right)$. The overall behavioral variance across all measures *M* (explained by either HEN or LEN) is then:

$$M=\frac{Tr(\sum_{c})}{Tr\left(\sum_{c}\right)+\;Tr\left(\sum_{e}\right)},$$

with Tr(.) being the trace operator. The explained variance for a single cognitive variable $M_{i}$ is computed as:

$$M_{i}=\frac{\sum_{c}\left(i,i\right)}{\left(\sum_{c}\left(i,i\right)+\sum_{e}\left(i,i\right)\right)}.$$

This results in one value between zero and one, across all measures, and for each behavioral measure. Since we account for covariates the model becomes:

Y=XB+C+E,

where X is the 97×5 matrix of covariates (Section 4.4), and B a 5×7 matrix of fixed effects (Ge et al., 2016). To remove the covariate matrix from the model, the data is projected onto a 97 – 5-dimensional subspace resulting in the transformed model:

$$\tilde{Y}=\tilde{C}+\tilde{E},$$

which is equivalent to the original model, see Ge et al. (2015) for details. Significance for the average variance across all cognitive variables was assessed with a *p*-value derived from a Wald-test (*p*-Wald), and complementarily by permuting the rows and columns of *R* (*p*-Perm) (Ge et al., 2016). The results described in 2.1, 2.2 were calculated by running the model separately for *RHEN* and *RLEN*, as well as for their alternatively derived (random) versions.

### 4.6 Software and code used in the analysis

aCompCor denoising was done with DPABI (Yan et al., 2016), which was developed in MATLAB (The MathWorks Inc., Natick, MA, US). Further preprocessing was done in Python with the help of the Nilearn toolbox (https://zenodo.org/records/10579570). Sliding-window analysis was done in Python with TENETO (https://zenodo.org/records/3626827) (Thompson et al., 2017). SampEn calculation was done in Python with EntropyHub (Flood & Grimm, 2021). Further statistical analyses were done in MATLAB with the help of the following toolboxes: Brain Connectivity Toolbox (https://sites.google.com/site/bctnet/home), statistics-resampling package (https://doi.org/10.5281/zenodo.3992392), and the BrainSpace toolbox (https://brainspace.readthedocs.io/en/latest/index.html) (Vos de Wael et al., 2020). Figures were in part created with Matplotlib (Hunter, 2007), MNE (https://doi.org/10.5281/zenodo.592483) (Gramfort et al., 2013), NiBabel (https://zenodo.org/records/10363247), and Connectome Workbench (Marcus et al., 2011). MATLAB code for the statistical model can be found here (people.csail.mit.edu/msabuncu/morphometricity).

## Ethics statement

For the HCP-EP data procedures were approved by the Partners Healthcare Human Research Committee/IRB and complied with the Declaration of Helsinki. Participants provided written informed consent, or in the case of minors, parental written consent, and participant assent.

## CRediT authorship contribution statement

**Fabian Hirsch:** Writing – review & editing, Writing – original draft, Visualization, Software, Methodology, Formal analysis, Conceptualization. **Ângelo Bumanglag:** Writing – review & editing, Software, Methodology, Formal analysis. **Yifei Zhang:** Writing – review & editing, Software, Methodology, Formal analysis. **Afra Wohlschlaeger:** Writing – review & editing, Supervision, Project administration, Methodology.

## Declaration of Competing Interest

The authors declare that they have no known competing financial interests or personal relationships that could have appeared to influence the work reported in this paper.

## Data Availability

Data will be made available on request.
