# Supplementary material for: Diverging functional connectivity timescales: Capturing distinct aspects of cognitive performance in early psychosis
Source: Neuroimage Clin. 2024 Aug 23;43:103657. doi: 10.1016/j.nicl.2024.103657 (PMC11401179; doi:10.1016/j.nicl.2024.103657)
Supplement: Supplementary Data 1 [file mmc1.docx]

**Appendix**

*Univariate model*

To obtain the results described in Sections 2.3 and 2.6.2 we used a univariate version of the multivariate model (Liegeois et al., 2019; Sabuncu et al., 2016):

$y=Xb+Wu+e$,

where *y* corresponds to a vector whose *N* entries contain the values of a given cognitive variable for all subjects, and *b* to a vector of fixed effects. $W$ is a column-standardized *N*×*P* matrix, with *P* corresponding to the total number of edges in the high-entropy network- (HEN)/low-entropy network- (LEN) template (Section 4.3 in the main manuscript), $u$ $\sim\mathcal{N}(0, \sigma_{c}/P)$ is a vector of random effects, and $e$ $\sim\mathcal{N}(0, \sigma_{e})$ the residual. *W* contains the subjects’ standardized ESE estimates for a given template, and assuming that the elements of *u* are independent, this model can be transformed to the model from Section 4.5: $\mathrm{Cov}(y) = \sigma_{c}\cdot R + \sigma_{e}\cdot I$, with $R = {W\cdot W}^{T}/P$. The squared entries of the best linear unbiased predictor of *u* ($\hat{u}$) (Yang, Lee, Goddard, & Visscher, 2011) are a scaled estimate of the variance explained by the corresponding edge across all individuals. We computed $\hat{u}^{2}$ for each cognitive variable and weighted it by the loading of that variable on the first principal component derived from the whole set of behavioral variables (Liegeois et al., 2019), see Ge et al. (2016) for the underlying rationale.

*Statistical inferences*

The significance of the results reported in Sections 2.1-2.2 was assessed by estimating confidence-intervals (CIs) after resampling, since the model output consist of just one value between zero and one for every dependent variable, as well as the average variance explained. We used two different resampling methods to ensure robustness of the ensuing CIs: Bias-corrected bootstrap CIs (BS-CIs) were calculated based on 1000 bootstrap samples and their width was Bonferroni adjusted for multiple comparisons (Manly, 2018). As a complementary approach, we employed a block-version of jackknife-resampling (JK) in combination with random subsampling: Briefly, the JK-CIs were computed based on 1000 subsets of size $N - d$of the original data, randomly sampled with replacement (Shao & Tu, 1995a). If $\sqrt{N} < d < N - 1$, this yields a consistent JK variance estimator for most underlying statistics, so we used *d* = 10 (Shao & Tu, 1995b).

The significance of the results reported in Section 2.3 was assessed with two different null-models that were derived from the results of the univariate analysis. For both HEN and LEN (respectively) these results can be represented by a graph with 116 nodes whose edges are weighted according to the average behavioral variance explained across variables. The binarized versions of these graphs are equivalent to the corresponding HEN/LEN template. For the first null-model we just randomly shuffled the edges (10000 iterations) before calculating the average variance explained at the system-level. However, we also wanted to know if there were certain networks (NWs) and/or NW-interactions that explained significantly more (average) behavioral variance than would be expected, based on the relative centrality and explanatory power of their constituent nodes in the underlying templates. To achieve this we created degree- and strength matched random NWs (10000 iterations) (Rubinov & Sporns, 2011), before calculating the average variance explained at the system-level.

*References*

Ge, T., Reuter, M., Winkler, A. M., Holmes, A. J., Lee, P. H., Tirrell, L. S., . . . Sabuncu, M. R. (2016). Multidimensional heritability analysis of neuroanatomical shape. *Nature Communications, 7*(1), 13291. doi:10.1038/ncomms13291

Liegeois, R., Li, J., Kong, R., Orban, C., Van De Ville, D., Ge, T., . . . Yeo, B. T. T. (2019). Resting brain dynamics at different timescales capture distinct aspects of human behavior. *Nat Commun, 10*(1), 2317. doi:10.1038/s41467-019-10317-7

Manly, B. F. (2018). *Randomization, bootstrap and Monte Carlo methods in biology*: chapman and hall/CRC.

Rubinov, M., & Sporns, O. (2011). Weight-conserving characterization of complex functional brain networks. *Neuroimage, 56*(4), 2068-2079. doi:10.1016/j.neuroimage.2011.03.069

Sabuncu, M. R., Ge, T., Holmes, A. J., Smoller, J. W., Buckner, R. L., & Fischl, B. (2016). Morphometricity as a measure of the neuroanatomical signature of a trait. *Proc Natl Acad Sci U S A, 113*(39), E5749-5756. doi:10.1073/pnas.1604378113

Shao, J., & Tu, D. (1995a). Computational Methods. In J. Shao & D. Tu (Eds.), *The Jackknife and Bootstrap* (pp. 190-231). New York, NY: Springer New York.

Shao, J., & Tu, D. (1995b). Theory for the Jackknife. In J. Shao & D. Tu (Eds.), *The Jackknife and Bootstrap* (pp. 23-70). New York, NY: Springer New York.

Yang, J., Lee, S. H., Goddard, M. E., & Visscher, P. M. (2011). GCTA: a tool for genome-wide complex trait analysis. *Am J Hum Genet, 88*(1), 76-82. doi:10.1016/j.ajhg.2010.11.011
